# Rapid Microfluidic Mixing Method Based on Droplet Rotation Due to PDMS Deformation

**DOI:** 10.3390/mi12080901

**Published:** 2021-07-29

**Authors:** Chunyang Wei, Chengzhuang Yu, Shanshan Li, Feng Pan, Tiejun Li, Zichao Wang, Junwei Li

**Affiliations:** 1 Hebei Key Laboratory of Robotic Sensing and Human-Robot Interactions, School of Mechanical Engineering, Hebei University of Technology, Tianjin 300132, China; 201811201008@stu.hebut.edu.cn (C.W.); 201921202075@stu.hebut.edu.cn (F.P.); 201931204017@stu.hebut.edu.cn (Z.W.); 2 State Key Laboratory of Reliability and Intelligence of Electrical Equipment, Hebei University of Technology, Tianjin 300130, China; 201811201004@stu.hebut.edu.cn; 3 Jiangsu Key Laboratory of Advanced Food Manufacturing Equipment and Technology, Wuxi 214122, China; 4 Institute of Biophysics, School of Science, Hebei University of Technology, Tianjin 300401, China; 5 Department of Computer Science and Electrical Engineering, Hebei University of Technology, Langfang 065000, China

**Keywords:** microfluidics, mixing, droplet rotation, PDMS deformation

## Abstract

Droplet-based micromixers have shown great prospects in chemical synthesis, pharmacology, biologics, and diagnostics. When compared with the active method, passive micromixer is widely used because it relies on the droplet movement in the microchannel without extra energy, which is more concise and easier to operate. Here we present a droplet rotation-based microfluidic mixer that allows rapid mixing within individual droplets efficiently. PDMS deformation is used to construct subsidence on the roof of the microchannel, which can deviate the trajectory of droplets. Thus, the droplet shows a rotation behavior due to the non-uniform distribution of the flow field, which can introduce turbulence and induce cross-flow enhancing 3D mixing inside the droplet, achieving rapid and homogenous fluid mixing. In order to evaluate the performance of the droplet rotation-based microfluidic mixer, droplets with highly viscous fluid (60% *w*/*w* PEGDA solution) were generated, half of which was seeded with fluorescent dye for imaging. Mixing efficiency was quantified using the mixing index (MI), which shows as high as 92% mixing index was achieved within 12 mm traveling. Here in this work, it has been demonstrated that the microfluidic mixing method based on the droplet rotation has shown the advantages of low-cost, easy to operate, and high mixing efficiency. It is expected to find wide applications in the field of pharmaceutics, chemical synthesis, and biologics.

## 1. Introduction

Microfluidics has attracted much attention in chemical analysis, pharmacology, biologic, and catalysis due to its easy integration, multi-function, and miniaturization [1,2,3,4]. Among the many applications, microfluidic mixing is crucial due to the need for homogenization of multiple reagents in biological or chemical reactions [5,6,7]. However, a well-known problem in microfluidic mixing is its efficiency due to its feature by laminar flow; thus, the mixing is dominated by molecular diffusion, which is time-consuming with low efficiency. For this reason, many methods have been studied to enhance the mixing to achieve rapid, homogenous mixture in lab-on-a-chip (LOC) systems [8,9,10].

The droplet-based mixing method is a powerful microfluidic mixing tool because of the internal recirculation and isolated environments. However, the three-dimensional (3D) flow only occurs within each half of the droplet if they only move in straight microchannels. Thus, the mass transfer in the transversal direction relies on natural diffusion, which shows low mixing efficiency. The secret to enhancing the mixing is the increase in the diffusive flux between the several fluids in a droplet mixer, involving parameters such as diffusion coefficient, concentration gradient, temperature, and contact area. There are active [11,12] and passive [13,14,15] droplet-based mixers generally used in mixing studies. Compared with the active mixers, which involve extra energy, the passive mixers achieve high mixing efficiency only by the droplet movement, showing several advantages in terms of (1) suitable stability: the droplet-based mixers are almost impossible to fuse due to the abandonment of electric or heat field, which is advantageous when the density of droplets is large even the droplet are next to each other; (2) bio-friendliness: active biomolecules such as DNA and enzymes can live in a milder environment and maintain their original properties without being affected by electric or heat field; (3) easy to operate: there is no multiphysics coupling but only flowing. Thus, the platform setup is concise, and the experiment is quite easy to operate. The most commonly used passive mixing method was droplet-deformation in serpentine microchannels [16,17], including stretch and fold, which can involve turbulence and induce cross-flow, thus achieving rapid and homogeneous mixing. In order to achieve the deformation, the droplet generation is in a squeezing regime [18], where droplets are filled almost the entire cross-section of the microchannel. Thus, the droplet generation frequency is low, meaning that there are few microfluids mixed per unit time. In addition, the long serpentine microchannels (usually more than a few centimeters) occupy large regions in a microfluidic chip, which is not friendly to the integration of multi-function modules and make miniaturization difficult. Lastly, the fabrication of such micro-device likely undergoes higher risks for failure due to the complex microchannel configurations. With the increasing prospect of droplet mixing in microfluidic applications, it is of great importance to developing a method that can achieve rapid mixing inside droplets without long serpentine channels.

Here in this work, a novel method based on droplet rotation is put forward to achieve rapid and homogeneous mixing in lab-on-a-chip (LOC) systems. The compatibility of PDMS (polydimethylsiloxane) channels with certain solvents is a well-known problem in microfluidics because of the porous nature of PDMS. In this study, we take advantage of this PDMS deformation [19,20,21] property. When an organic solvent penetrates the PDMS, a rubber-like expansion occurs in the material, resulting in geometric deformation on the roof of the microchannel. Hexadecane served as the organic solvent, and it was injected into the microchannel to achieve the subsidence of the channel roof, which may increase the flow resistance and strongly affect the trajectories of droplets by pushing them away from the centerline. At the same time, due to the non-uniform distribution of the flow field in the microchannel, the droplets will be subjected to a torsion moment that can result in rotating, thus inducing the cross-stream between the two halves of droplets to achieve rapid mixing.

## 2. Materials and Methods

### 2.1. Chip Fabrications

**Figure 1:**
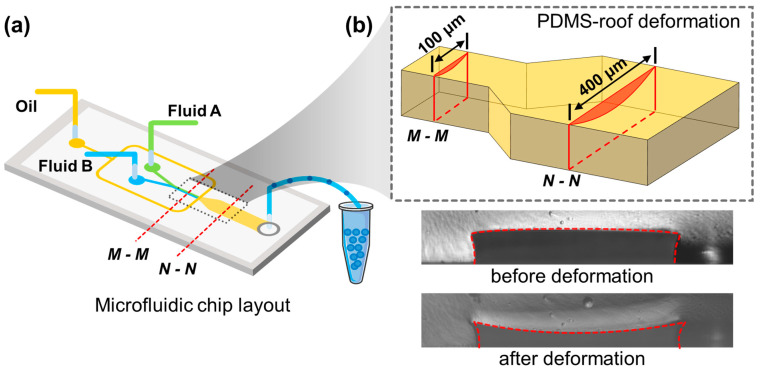
Schematic of experiment platform. (**a**) Oil, fluid A, and fluid B are introduced into the microchannel separately. Droplets are generated, mixed, and then collected into a centrifuge tube. (**b**) The enlarged image shows the subsidence of the channel roof at 100 μm width and 400 μm width. The two pictures below demonstrate the cross-sections of the microchannel before and after deformation.

To fabricate the droplet rotation-based mixing platform shown in Figure 1a, a SU-8 (Microchem, Westborough, MA, USA) mold insisting of inlets and microchannels was firstly fabricated by single lithography technology. The microchannels have a nominal height of $h_{c}$ = 50 μm and widths w vary from 100 to 400 μm. Then, PDMS base and curing agents (Sylgard 184 Silicone elastomer kit, Dow Corning) were well mixed (12:1% *w*/*w*) and degassed to remove air bubbles. To note, the ratio of PDMS base to curing agents should be greater than 10:1 to obtain a softer elastomer that is easily deformed. Then, the PDMS polymer was cast onto the defined structure in SU-8 mold and cured at 80 °C for 2 h. The inlets were punched by a 1 mm diameter biopsy puncher (Suzhou Wenhao Co. Ltd., Suzhou, China). Then the PDMS replica was bonded to a borosilicate glass slide (25 × 75 × 1 mm) after oxygen plasma treatment at 80 W power for 40 s (PDC-MG, Suzhou Wenhao Co. Ltd., Suzhou, China). After the PDMS and bottom glass were plasma bounded, hexadecane was driven into the microchannels and encapsulated for 10 min to induce subsidence of the channel roof. Next, for generating water-in-oil droplets successfully, Rain-X (PPG Industries, Chicago, IL, USA) was driven into the microchannels and encapsulated for 2 min to make the wall hydrophobic. Lastly, the microchannels were rinsed with ethanol for 2 min and heated at 100 °C to dry inside.

### 2.2. Experimental Setup

The schematic description of the droplet rotation-based platform is shown in Figure 1. As shown in Figure 1, there are three inlets for fluid A (60% *w*/*w* PEGDA solution with fluorescent molecules), fluid B (60% *w*/*w* PEGDA solution), and the oil, respectively, the droplets were generated, mixed, and then collected into a centrifuge tube. As demonstrated in the enlarged view, the core part of the microfluidic chip is the droplet rotation region with a channel width of 400 μm.

The experimental setup consists of a microfluidic chip, a fluid pumping unit (ELVE FLOW OB1 MK3, Paris, France), and an inverted microscope (Nikon Eclipse TI 2, Tokyo, Japan) equipped with a CCD camera (Nikon DS-QI2, Tokyo, Japan). Droplets were generated using a flow-focusing generator. The dispersed phase consists of 60% *w*/*w* PEGDA solutions (EFL, Suzhou, China) seeded with or without fluorescent molecules. The purpose of fluorescent dye is to demonstrate the mixing efficiency of the droplet rotation-based microfluidic mixer.

These two streams of PEGDA solutions were driven by one single air source to ensure they are driven at the same flow rate; thus, they flowed side by side before reaching the junction and then pinched off into droplets half by half. Emulsion droplets were stabilized using 5 wt.% polyglycerol polyricinoleate (Lingbao Techltd, Beijing, China) as a surfactant in the FC40 oil phase. Sodium fluorescein (FuChen, Tianjin, China) was used as a fluorescent dye to seed one of the flow streams.

### 2.3. Data Recording and Process

The mixing efficiency can be indicated by the mixing index (MI), which is influenced by many factors, including fluid properties and operating conditions, such as the fluid velocity, fluid viscosity, interfacial tension, diffusion coefficient, temperature, and so on. The microscopic images were processed with the help of ImageJ (National Institute of Health, Bethesda, MD, USA). MI is calculated based on the following equation [22]:

(1) $$MI=1-\frac{\sqrt{\left(\frac{1}{n}\right)\sum {\left(I_{i}-I_{av}\right)}^{2}}}{I_{av}}$$

where $I_{i}$ is the fluorescent intensity of the selected point, $I_{av}$ is the average intensity, and *n* is the number of selected points.

## 3. Results and Discussion

### 3.1. Fluorescence Images of Mixing Caused by Droplet Rotation

**Figure 2:**
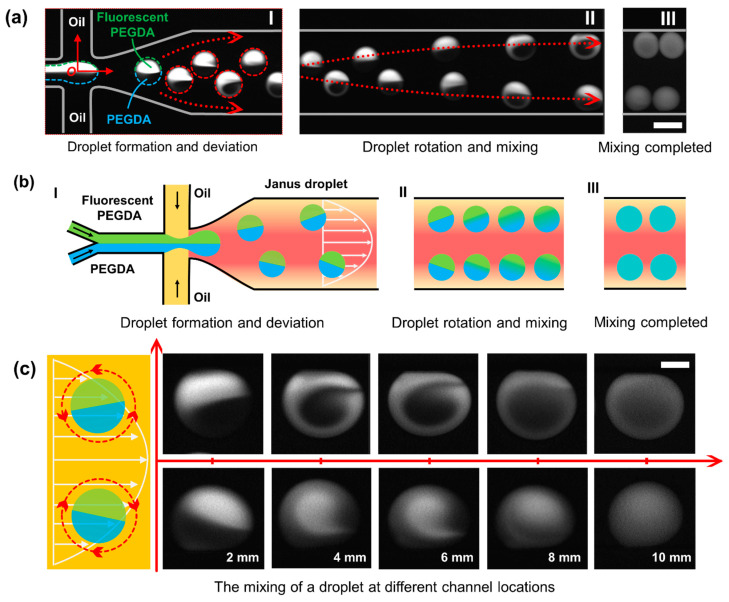
Fluorescence images obtained through the microscope and CCD camera. (**a**) **I**: Droplet formation and trajectory deviation (red dash line). **II**: Droplet rotation and mixing. **III**: Droplet mixing completed. Scale bar is 100 μm. (**b**) Schematics of the droplet generation and droplet mixing. (**c**) The rotating droplets at different positions. Scale bar is 40 μm.

As shown in Figure 2(aI), after all the regents were introduced into the microchannel independently and met in the junction, Janus droplets were formed with the help of a combination of Y-channel and flow-focusing design. In general, the two regents from the Y-channel form the dispersed phase, and the oil served as the continuous phase (incompatible with the dispersed phase). The dispersed phase was sheared by the continuous phase and pinched off to form droplets due to the special geometric design. With the help of a microscope and CCD camera, there shows clear boundaries between the two halves inside the droplet.

When droplets were generated and then traveled in a straight microchannel, two symmetric counter-rotating recirculation zones were formed in each half of the droplet [23,24]. Thus, there will be no external disturbance, creating a barrier limiting the mixing between the two halves. Only when the two halves are agitated can rapid and complete mixing be achieved. The crucial element and main trigger of the microfluidic mixing, herein, is the rotation behavior, which can induce cross-flow and destroy the barrier. In this design, while the droplets moved along the centerline without trajectory deviation in the initial movement right-side the cross junction, they were pushed away from the centerline when entered the collapsed region and rotate due to the non-uniform distribution flow field, thereby introduce turbulence and destroy the equilibrium interface between the two fluids [25,26]. The disordered 3D fluid behavior inside the droplet makes the contact area between the two fluids gradually increase, thus enhance the diffusive flux. As shown in Figure 2(aII,aIII), with the continuous rotation of the droplet, this mixing behavior continues until the mixing is complete. Figure 2b shows the schematics of the droplet generation and droplet mixing, which is corresponding to Figure 2a.

As shown in Figure 2c, the distribution of the flow field is non-uniform, and the flow velocity is parabolic along the transverse direction of the microchannel. When the droplet is located in the channel center, the two halves of the droplets are balanced, and no rotation occurs. However, when the droplet deviates away from the centerline, the two halves of the droplet are not uniformly stressed, and the rotation occurs. Duo to the symmetry of the flow field, the upper droplets rotated counterclockwise while the lower droplets rotate clockwise. The rotation introduced turbulence and destroyed the barrier between the two inner liquids, which increased the contact area and increased the diffusion flux. The series of pictures right-side shows the mixing state of the droplet at different positions (a corresponding video is provided in Supplementary Materials Video S1).

In fact, the rotating mechanism is a complex phenomenon involving the understanding of non-uniform flow field, non-uniform mass distribution between the two halves within the droplet, and their strong dynamic coupling, which is beyond the scope of this study. For example, the subsidence of the channel roof will affect the distribution of the flow field, transforming it from a two-dimensional parabolic distribution to a three-dimensional complex distribution, resulting in the 3D rotation of the droplet. Other disturbances seemingly weak can also affect the direction and speed of rotation, such as the small difference in density or viscosity between the two halves of the droplet.

**Figure 3:**
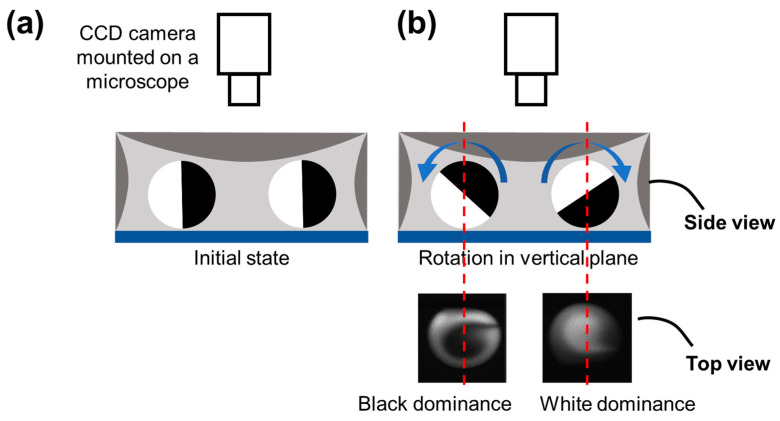
Schematics of droplet rotation. (**a**) The black and white areas were balanced when there is no rotation behavior in the vertical plane. (**b**) There would be one color covering the central region from the top view when there exists rotation behavior in the vertical plane.

When the droplet rotation occurs only in the horizontal plane, each of the two fluids (characterized by black and white) occupies half of the droplet from the top view. However, as shown in Figure 2c, from the real-time pictures taken from the top view, the half-half status of the two fluids in a rotating droplet changed gradually to form a Taiji pattern. Thus, there must be a rotation behavior that occurred perpendicular to the horizontal plane. As shown in Figure 3, the rotation in the vertical plane will result in black or white dominance in the droplet from the top view, exactly matching the real-time fluorescent pictures.

In order to shed light on the mixing mechanism, the study only focused on the droplet rotation on two-dimensional as discussed in Section 3.3, which is the largest component in the 3D rotation of the droplet.

### 3.2. Droplet Rotation Caused by PDMS Deformations

The compatibility of PDMS channels with certain solvents is bad due to the porous nature of PDMS. Studies have shown the secret to realize the PDMS deformation is the use of organic solvents such as hexadecane, and silicone oil, which can penetrate the PDMS and result in a rubber-like expansion. Here in this work, solvent hexadecane was encapsulated in a microchannel for 10 min to obtain subsidence on the roof of the microchannel.

The cross-linked PDMS consists of high-molecular-polymers; when PDMS is immersed in the organic solution, the small solvent molecules continuously diffuse into the polymer chains and make it stretched. As the network continues to stretch, there is also a tendency to restore its original state due to the entropy elasticity. Thus, when the solvation and entropy elasticity keep balance, the swelling process can reach equilibrium, and the PDMS deformation is completed. The “PDMS polymer-solvent” system satisfies the following equation [27,28]:

(2) $$\frac{\rho V_{m,1}}{\bar{M_{c}}\left(\frac{1}{2}-\chi \right)}=\Phi^{\frac{5}{3}}$$

where ρ is polymer density, $V_{m,1}$ is the molar volume of the solvent, $\bar{M_{c}}$ is the average molecular weight between two crosslink points of PDMS, χ is the interaction parameter between solute and solvent molecules, Φ is the volume fraction of solute, while it is also meaningful in terms of geometry. The solvents in the solution will penetrate the PDMS polymer; thus, the original dry PDMS polymer network will swell, resulting in an arched deformation. Φ can also be described by the following equation:

(3) $$\Phi =\frac{V_{PDMS}}{V_{PDMS}+V_{solvent}}=\frac{V_{PDMS}}{V_{Polymer-solvent\;}}$$

(4) $$V_{PDMS}=wh_{s}l$$

(5) $$V_{Polymer-solvent\;}=w\left(∆h+h_{s}\right)l$$

where $h_{s}$ is the depth of penetration, ∆h is the subsidence of the channel roof, *w* is the channel width, *l* is the channel length.

Based on the Equations (2)–(5), ∆*h* can be calculated by the following equation:

(6) $$∆h=h_{s}[{\left(\frac{\rho V_{m,1}}{\;\bar{M_{c}}\left(\frac{1}{2}-\chi \right)}\right)}^{-\frac{3}{5}}-1]$$

Only if the ∆h is large enough can the droplets be pushed away from the centerline; thus, it is crucial to create enough roof subsidence. Among the several parameters that affect ∆h, as shown in Equation (6), $\bar{M_{c}}$ is the easiest to change by adjusting the ratio of PDMS base to curing agents. When the ratio increased, the average molecular weight between two crosslink points increased; thus, the organic solvent could penetrate PDMS more easily and make ∆h increase. From a macro view, by reducing the proportion of curing agents, the resulting elastomer is softer and less rigid, which is more prone to deformation. While the higher ratio between the prepolymer to the curing agent enhances the roof subsidence, it may also result in prolonged curing time (when the ratio is 15:1, it takes 5 h to be cured completely at 80 °C). Moreover, a higher ratio may lead to unexpected and excessive subsidence of PDMS and increase the flow resistance significantly, even block the microchannels. As a result, it may fail to produce or deliver droplets within the microchannel. In our conditions, we optimized the PDMS/curing agent ratios as 12:1 to achieve excellent performance for droplet rotation and mixing with a reasonable curing time and a proper size of roof subsidence. As shown in Figure 1b, the deformations of the roof of PDMS microchannels can be experimentally measured by the optical method, which was 19.6 μm at the middle position from measurement. The deformation on the roof is much greater than that of the sidewall. The deformation of the two sidewalls is symmetrical; thus, the impacts on mixing caused by sidewall deformation were offset with each other; thus, it can be ignored herein. There is no deformation at the glass substrate, while the PDMS roof shows large deformation. That is to say, the roof subsidence is the most crucial factor for droplet rotation and the mixing performance within the droplets. Moreover, the mixing experiment carried out in this work also demonstrates that we can achieve droplet rotation and mixing by controlling the roof subsidence only. From these two points above, it is not necessary to pay attention to the effects of the deformation of sidewalls on the droplet rotations.

### 3.3. Effects of Contact Areas on Mixing Efficiency

**Figure 4:**
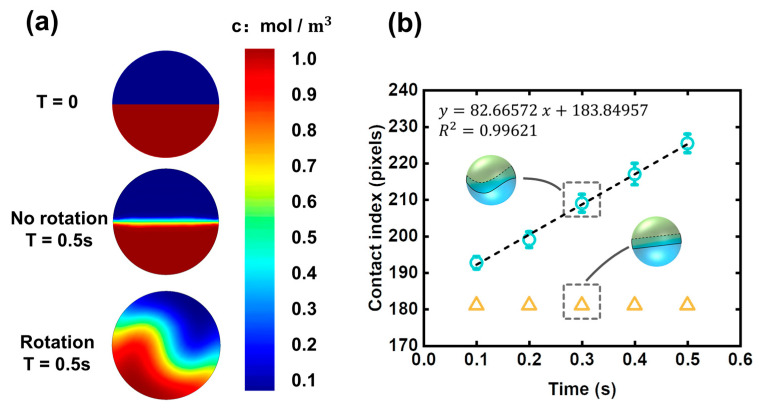
Contact areas between the two halves of the droplet. The SD represents the discrete of three curves that were used to represent the contact index. (**a**) The initial state, non-rotating state, and rotating state of the two halves. (**b**) The time dependence of contact index in rotating and non-rotating droplets.

The mixing state of the two halves of a droplet was shown in Figure 4a, which is a numerical prediction using commercial software, COMSOL Multiphysics. The multi-physical field is a combination of laminar flow (spf) and transport of diluted species (tds). The frozen rotor method was used the simulate the mixing process inside the droplet. The droplet diameter was set at 100 μm.

In the laminar flow part, rotation speed was set at 2π rad/s. The following equation was applied to the model.

(7) ρ(u·∇)u=∇·[−pI+K]+F

(8) ρ∇·u=0

(9) $$K=\mu (\nabla u+{\left(\nabla u\right)}^{T})$$

In the transport of diluted species part, the concentrations of the two fluids were set at 0 and 1 mol/m^3^ the droplet diffusion coefficient was set at $1\times 10^{-9}$ [m^2^/s]. The following equation was applied to the model.

(10) $$\nabla \cdot J_{j}+u\cdot \nabla_{cj}=R_{j}$$

(11) $$J_{j}=-D_{j}\nabla_{cj}$$

It can be seen that the diffusion flux is high in the rotating droplet while weak in the non-rotating droplet. Although the simulation here is two-dimensional, it can still reflect how the rotation affects the two halves of a droplet. The working principle of the droplet rotation is “increasing contact area”. Rotation behavior induces disturbance to the flow field inside the droplet, which can enhance cross-stream mixing, thus resulting in the increasing of contact area between the two halves of the droplet. Contact area has a crucial influence on the diffusion flux; herein, the contact area is indicated by the contact index, which can be obtained by measuring the length of the two-half boundary. Time dependence of contact index in rotating and non-rotating droplets was shown in Figure 4b. The contact index in a rotating droplet is greater than that of the non-rotating droplet, indicating there are more complicated entanglements between the two inner fluids when rotation behavior occurs.

The diffusion coefficient of fluorescent molecules in high-viscosity solution (such as PEGDA solution used here) is smaller compared with low-viscosity solution (such as pure water). Moreover, low-viscosity fluids are easier to be driven and merge with each other; thus, it can be inferred that there will be a better mixing effect for the experiments involving low-viscosity fluids.

**Figure 5:**
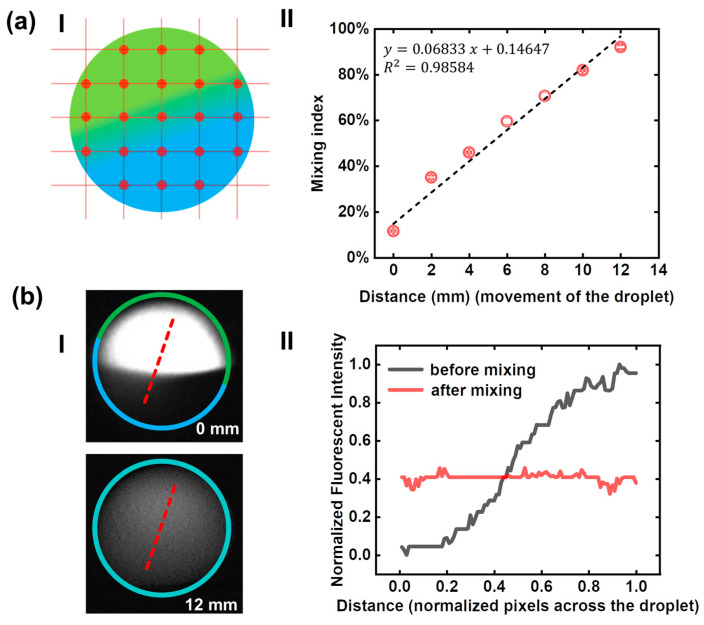
Calculation of mixing index (MI). (**a**) **I**: There were twenty-one points selected in each fluorescent image for intensity analysis, which can be used to calculate the mixing index (MI). **II**: Mixing index of droplets at different traveling distances. (**b**) **I**: Two dash line scans across the microphotographs of the unmixed and mixed droplets. **II**: The distribution of fluorescence intensity of the rotating droplet is uniform compared with the initial state.

In order to study the mixing efficiencies at different positions, fluorescent images were taken from seven locations (0, 2, 4, 6, 8, 10, 12 mm) were used for fluorescent intensity analysis. As shown in Figure 5a, we took 21 points in each microscopic to calculate the mixing index. The corresponding data graph showed the continuous improvement of the mixing efficiency as the droplet traveling on. The X-axis is the distance that the droplet traveled after entering the rotation region (the center of the junction where generates the droplet is marked as x = 0 as shown in Figure 2a).

In order to evaluate the mixing effect in general, we studied the mixing state of the droplet at the starting point and the endpoint. As shown in Figure 5b, the initial state and mixed state of a droplet were presented. When the droplet rotated for a period of time, the clear boundaries between the black and white area were gone, and the two halves were thoroughly mixed. Fluorescent intensity profiles were measured from the microscopic images. Two dash line scans across the droplet demonstrate the intensity profiles before and after mixing. From the right-side graph, it can be seen that the fluorescence intensity of the mixed droplet is relatively homogeneous compared with the unmixed droplet, indicating that the mixing based on droplet rotation is achievable and effective.

### 3.4. Effects of Flow Rates on Mixing Efficiency

In general, when the generated droplets move forward inside a straight microchannel without any perturbation, the cross-stream mixing is suppressed due to the barrier between the two halves of the droplet, which can prevent the cross-stream mixing. At the same time, the chaotic advection inside the two halves cannot guarantee a full mixing. However, when placed in a non-uniform distributed flow field, the droplet can rotate, and the barrier between the two halves is broken. In fact, there are still many factors that affect the mixing efficiency, including the fluids density, fluids viscosity, diffusion coefficient, contact area, concentration gradient, and so on.

**Figure 6:**
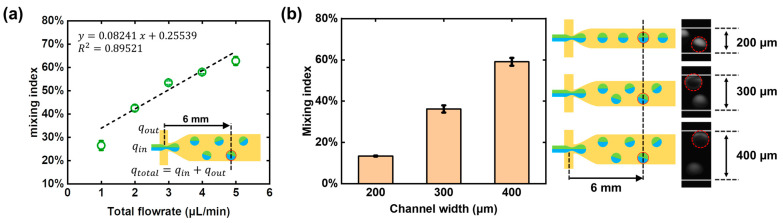
Other factors affecting the mixing index including “total flow rate” and “channel width”. (**a**) Flow rate dependence of mixing index. Each standard deviation under different flow rates was obtained from three independent experiments in the same microfluidic chip. (**b**) The effect of channel width on mixing index. Each standard deviation under different channel width was obtained from three independent experiments at the same flow conditions. The real-time pictures right-side had shown the different mixing states in these three kinds of microchannels.

We have also studied the effect of the fluid flow rate on the mixing index. Since the rotation of droplets are affected by the non-uniform distribution of the flow field, the total flowrate $q_{total}$ consisting of $q_{in}$ (water phase) and $q_{out}$ (oil phase) is also discussed. As shown in Figure 6a, the mixing efficiency of the droplets at the 6 mm position of the microchannel under different flowrate conditions were studied, respectively. The $q_{total}$ was kept at 1 μL/min ($q_{in}-q_{out}:0.4-0.6$), 2 μL/min ($q_{in}-q_{out}:0.8-1.2$), 3 μL/min ($q_{in}-q_{out}:1.2-1.8$), 4 μL/min ($q_{in}-q_{out}:1.7-2.3$), and 5 μL/min ($q_{in}-q_{out}:2.1-2.9$), so as to generate droplets of uniform size (CV < 5%). As the fluid flow rate increased, the flow field changed more drastically along the transverse direction of the microchannel, the force acting on the upper and lower parts of the droplet was more unbalanced, so the torsional moment increased, thus resulting in the more intense rotation and larger diffusion flux.

### 3.5. Effects of Channel Widths on Mixing Efficiency

The PDMS deformation has a crucial effect on the droplet rotation-based mixing. The subsidence of the channel roof is not enough when the PDMS deformation is insufficient; thus, the droplets will not be pushed away from the centerline, and there will be no rotation, resulting in low diffusion flux. From the above analysis in Section 3.2, it has been demonstrated that the ratio of PDMS base and curing regents can affect the PDMS deformation. By reducing the amount of curing agent used, a softer PDMS elastomer can be obtained, and more deformation can be achieved. At the same time, the roof subsidence can also be effectively controlled by the microchannel design. As shown in Figure 6b, three microchannels with the width of 200, 300, and 400 μm were used in the droplet rotation-based mixing experiments, and the fluorescent images at the same travel distance (6 mm) were taken for the intensity analysis. When the channel width reduced from 400 μm to 200 μm, the mixing index changed from 59% to 13%, showing a drop in the mixing efficiency. This phenomenon could be explained in geometrics. The roof subsidence in a narrow microchannel is so small that the flow resistance cannot increase significantly; thus, the droplet cannot be placed in a sufficiently non-uniform distributed flow field, and the chaotic advection in a rotating droplet will not happen. In general, the wider the microchannel, the greater the subsidence and the more impact on the droplet trajectory.

## 4. Conclusions

In this study, we demonstrated the mixing capability of droplet rotation integrated with PDMS swelling as a proof of concept. In order to analyze the mixing process of a rotating droplet, a series of microscopic images were taken for fluorescence analysis. The quantitative evaluation of the mixing index and the effect of the flowrate and microchannel width on the mixing index were also studied. With the help of fluorescent imaging, when the mixing index is higher than 0.8, the mixing can be considered acceptable. As the rotation behavior keep on, the mixing index will increase indefinitely to 100%; however, by optimizing various types of parameters, rapid and homogenous mixing can be achieved in a shorter time or less traveling with a high mixing index. The droplet rotation-based mixing system demonstrated here is easy to operate, and the device is concise with no complicated microstructure, providing an effective method for fluid mixing in many lab-on-chip applications. For example, nitrifying is a dangerous process due to the fast reaction and the huge heat dissipation. In the traditional kettle reaction, the reactor is large, and the heat fluctuations in the system are difficult to regulate, easily resulting in an explosion. By means of the mixing method proposed herein, the chemical reaction can be achieved using very few reagents, greatly increasing the safety of the reaction. At the same time, each droplet is a separate reaction chamber, which can achieve large-scale parallel experiments. This unique approach of using PDMS deformation to realize droplet rotation and mixing shows great prospects in the field of chemical analysis, pharmacology, and biological research.

## Supplementary Materials

The following are available online at https://www.mdpi.com/article/10.3390/mi12080901/s1, Video S1: Droplet rotation and mixing in a microchannel.
